# Establishment of a Chinese critical care database from electronic healthcare records in a tertiary care medical center

**DOI:** 10.1038/s41597-023-01952-3

**Published:** 2023-01-23

**Authors:** Senjun Jin, Lin Chen, Kun Chen, Chaozhou Hu, Sheng’an Hu, Zhongheng Zhang

**Affiliations:** 1 Emergency and Critical Care Center, Department of Emergency Medicine, Zhejiang Provincial People’s Hospital, Affiliated People’s Hospital, Hangzhou Medical College, Hangzhou, Zhejiang 310014 China; 2 Department of Critical Care Medicine, Affiliated Jinhua Hospital, Zhejiang University School of Medicine, Jinhua, China; 3 Department of Emergency Medicine, Key Laboratory of Precision Medicine in Diagnosis and Monitoring Research of Zhejiang Province, Sir Run Run Shaw Hospital, Zhejiang University School of Medicine, Hangzhou, 310016 China

**Keywords:** Infectious diseases, Health policy

## Abstract

The medical specialty of critical care, or intensive care, provides emergency medical care to patients suffering from life-threatening complications and injuries. The medical specialty is featured by the generation of a huge amount of high-granularity data in routine practice. Currently, these data are well archived in the hospital information system for the primary purpose of routine clinical practice. However, data scientists have noticed that in-depth mining of such big data may provide insights into the pathophysiology of underlying diseases and healthcare practices. There have been several openly accessible critical care databases being established, which have generated hundreds of scientific outputs published in scientific journals. However, such work is still in its infancy in China. China is a large country with a huge patient population, contributing to the generation of large healthcare databases in hospitals. In this data descriptor article, we report the establishment of an openly accessible critical care database generated from the hospital information system.

## Background & Summary

Critically ill patients managed in the intensive care unit (ICU) are usually monitored closely for organ dysfunctions, and are treated intensively by a variety of supportive modalities^1,2^. Vital signs, laboratory tests, and medical treatments were obtained at a higher frequency than those treated in the general ward. Such daily intensive management will produce a huge amount of information including medical orders, imaging studies, laboratory findings, and waveform signals. The data generation mechanisms may reflect key factors related to the healthcare system, the pathophysiology of underlying disease, and patient’s preferences and cultures^3^. Thus, in-depth data mining of such large databases, such as risk factor analysis, predictive analytics, and causal inference^4–6^, can provide more insights into clinical research questions. More knowledge or pearls of wisdom can be obtained from data mining, and the translation of the knowledge into clinical practice may potentially improve clinical outcomes^7,8^.

Most published scientific reports do not make their original raw data freely accessible in the current critical care research community, partly attributable to confidentiality issues. The unwillingness to share data makes it difficult to reproduce the reported results. Furthermore, the exploration of a such large database from a single research group could be biased and limited. Thus, strenuous efforts have been made to encourage the scientific community to share their raw data, which is also supported by the open data campaign^9,10^. Several openly accessible critical care databases have been established, mainly reflecting the healthcare systems of western countries^11–13^. China is a large country with a huge patient population. For example, the estimated incident sepsis cases are about 3 million in 2017, accounting for nearly 10% of the global incident cases^14^. Chinese hospitals also have special hospital information systems that are distinct from those of western countries. However, hospital information systems in Chinese hospitals are mainly used for clinical practice and are far less developed for research purposes. Data sharing is still in its infancy in the Chinese critical care community, which significantly impairs the transparency of scientific work and international collaborations. To the best of our knowledge, there are two critical care databases being established in China which focus on pediatric critically ill patients and those with infections^15,16^. Here, we reported the establishment of a large critical care database comprising high-granularity data generated from the information system of a tertiary care university hospital. Details of the database are reported in the paper to encourage new research through secondary analysis of the database.

## Methods

### Study setting and population

The study was conducted in Zhejiang Provincial People’s Hospital, Zhejiang, China from January 2012 to May 2022. All patients admitted to the ICU of the hospital were eligible. There were two ICUs in the hospital: one was the comprehensive central ICU and the other was the emergency ICU (EICU). There was no exclusion criterion in enrolling subjects because we believed that patients who were excluded by a particular study might be eligible for another study. Thus, we included all records in the information system related to ICU stays. The study was approved by the ethics committee of Zhejiang Provincial People’s Hospital (approval number: QT2022185). Informed consent was waived as determined by the institutional review board, due to the retrospective design of the study. The study was conducted in accordance with the Declaration of Helsinki.

### Database structure and development

The database is distributed as comma-separated value (CSV) files that can be imported to any relational database system. Each file contains a single table which will be further explained in the subsequent sections. Each individual subject can be identified by a series number (patient_SN) with the combination of digits and letters such as “3c74cf74c36241b7082ec35e458279dc”. Each unit hospital stay is denoted by a *Hospital_ID* with examples such as “9432117” and “336688072433”. The unique ICU stay can be identified by the *HospitalTransfer* table, which contains intrahospital transfer events for the subjects. All tables use *Hospital_ID* to identify an individual hospital stay, and the *HospitalTransfer* table can be used to determine ICU stays linked to the same patient and/or hospitalization.

We recommend the R package *tidyverse* for the management of the relational database because of its capability to streamline the workflow from data management to statistical analysis and to the training of machine learning models^17^. For large files, we recommend the *data.table* package to process the tabular data.

### Deidentification

All tables are deidentified according to the Health Insurance Portability and Accountability Act (HIPAA). All protected information is removed including addresses, date of birth, date of hospital admission, date of discharge, date of medical order, personal numbers (e.g. name, phone, social security, and hospital number), exact age on admission (age is discretized into bins). When creating the dataset, patients were randomly assigned a unique identifier (patient_SN and hospital_ID) and the original hospital identifiers were not retained. As a result, the identifiers in the database cannot be linked back to the original, identifiable data. All doctor/nurse/pharmacist identifiers have also been removed to protect the privacy of contributing providers.

## Data Records

The database comprises 8180 unique hospital admissions for 7638 individual patients from January 2012 to May 2022 and is available at the PhysioNet repository^18^. Table 1 shows the baseline demographics of hospital admissions. There are 2965 female and 5215 male patients in the dataset. The length of hospital days was 17 days (Q1 to Q3: 10 to 28). Male patients showed slightly longer hospital stay.

**Table 1:** Demographics and discharge status of the 8180 hospital admissions in the database.

Variables	Total (n = 8180)	Female (n = 2965)	Male (n = 5215)	p
Age_cut, n (%)				< 0.001
(0, 18]	35 (0)	14 (0)	21 (0)	
(18, 30]	272 (3)	100 (3)	172 (3)	
(30, 40]	493 (6)	167 (6)	326 (6)	
(40, 50]	695 (8)	204 (7)	491 (9)	
(50, 60]	1435 (18)	477 (16)	958 (18)	
(60, 70]	1738 (21)	624 (21)	1114 (21)	
(70, 80]	1674 (20)	613 (21)	1061 (20)	
(80, 90]	1310 (16)	558 (19)	752 (14)	
(90, 150]	528 (6)	208 (7)	320 (6)	
DaysHospitalStay, Median (Q1,Q3)	17 (10, 28)	16 (10, 26)	18 (10, 28)	< 0.001
StatusOnDischarge, n (%)				0.901
Cured	5666 (73)	2050 (73)	3616 (73)	
Dead	438 (6)	157 (6)	281 (6)	
Not cured	1202 (16)	437 (16)	765 (15)	
Unknown	444 (6)	153 (5)	291 (6)	

**Fig. 1:**
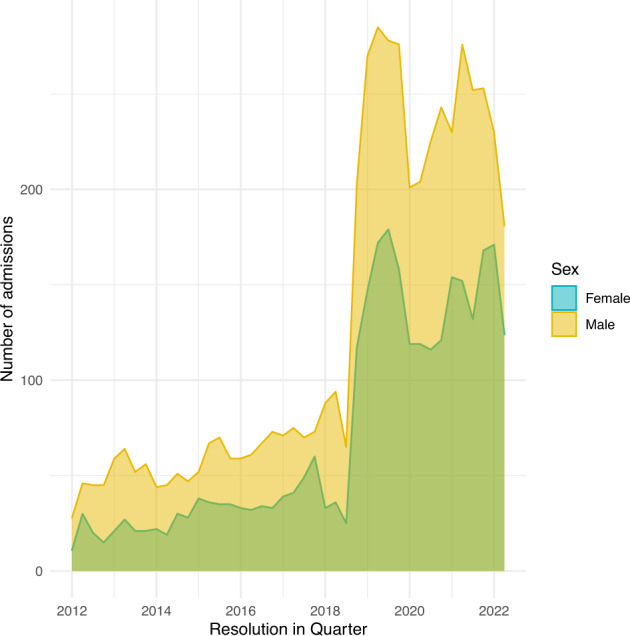
The number of admissions from the year 2012 to 2022.

**Fig. 2:**
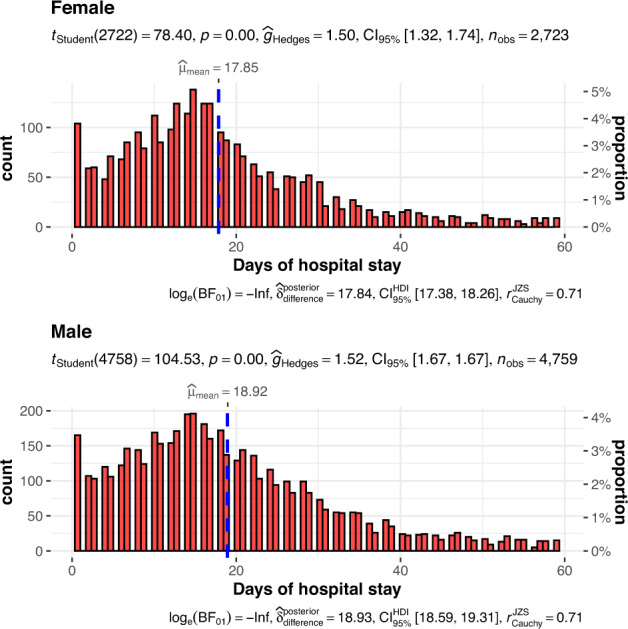
The distributions of hospital length of stay in male and female patients.

The number of hospital admissions for ICU patients increased remarkably after the year 2018 because of the expansion of bed numbers this year for both comprehensive ICU and emergency ICU (Fig. 1). The distributions of hospital length of stay are shown in Fig. 2, restricting to patients with a length of stay (LOS) <60 days.

**Fig. 3:**
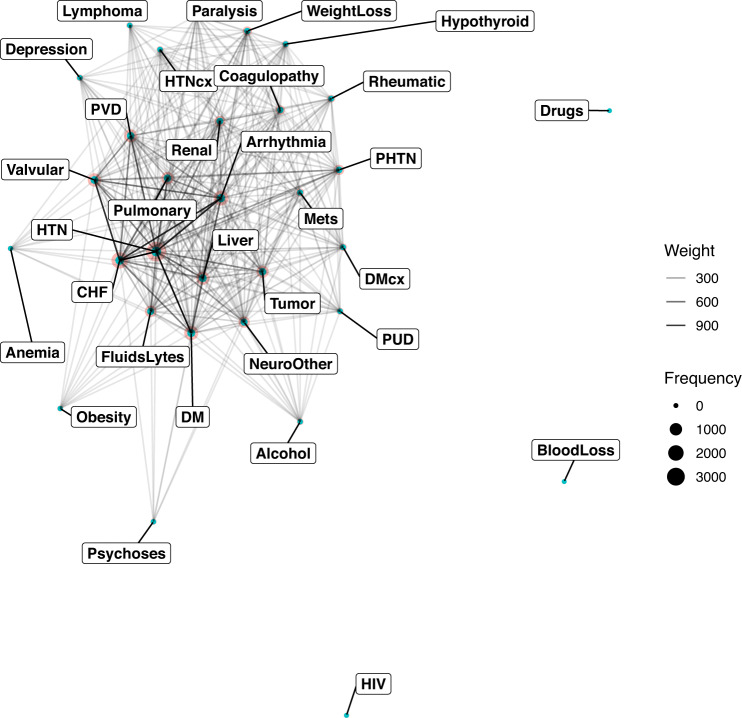
The co-occurrence network shows the frequency of diagnosis categories in the datasets. The size of the circle represents the number of diagnoses, and the transparency of the lines represents the frequency of coexisting. Abbreviations: PUD = Peptic ulcer disease; DM = Diabetes without chronic complication; DMcx = Diabetes with chronic complication; PVD = Peripheral vascular disorders; CHF = Congestive heart failure; HTN = Hypertension; HTNcx = Hypertension, complicated; PHTN = pulmonary hypertension; Mets = Metastatic solid tumor.

**Fig. 4:**
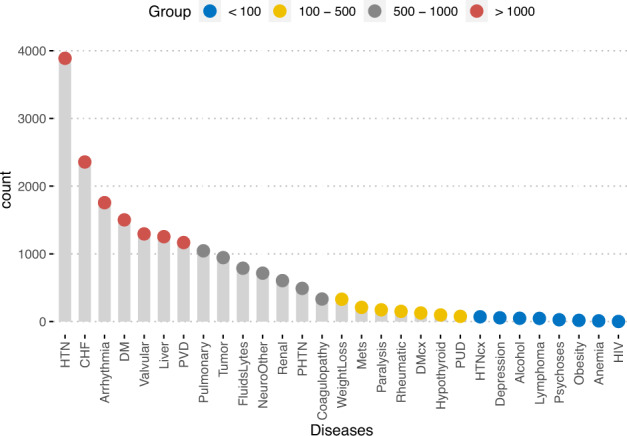
Dot chart showing the frequency of commonly encountered diseases in the dataset. Abbreviations: PUD = Peptic ulcer disease; DM = Diabetes without chronic complication; DMcx = Diabetes with chronic complication; PVD = Peripheral vascular disorders; CHF = Congestive heart failure; HTN = Hypertension; HTNcx = Hypertension, complicated; PHTN = pulmonary hypertension; Mets = Metastatic solid tumor.

We then categorized specific diagnoses into 31 categories to explore the characteristics of the population in the dataset^19^. The co-occurrences of the diseases are shown in Fig. 3. The results showed that pulmonary diseases are among the most common reasons for admission, followed by chronic heart failure (CHF). CHF usually coexists with valvular disorders. It is also noted that pulmonary diseases usually coexist with cardiac arrhythmia (Fig. 3). Figure 4 shows the frequency of these diseases. Hypertension is among the highest diseases in the study population, followed by chronic heart failure and arrhythmia.

### Classes of data

The data are organized into tables. There are a total of 17 tables comprising patient demographic data, medical order, laboratory findings, image studies, microbiology and hospital transfer events (Table 2). We will provide more details for each individual table to promote the reuse of our database.

**Table 2:** A general description of the tables in the database.

Table name	MD5_hashes	No. of rows	Description
Diagnosis.csv	3f838169d4655ee4fded3b85e812f7d0	143420	Diagnosis
DrugSens.csv	ddf274691afd3a092aea0de2c121a510	734024	Sensitivity to antibiotics for cultured bacteria
ExamReport.csv	59124429ba43e9d5d5a93d9b9f6565fb	93466	Examination report including CT, ultrasound and MRI
FirstNote.csv	05ef17211c6b1b193b3bf1aa6a55ebdd	6016	First progress note recorded on admission
HospitalTransfer.csv	3780660ae949c16f99e4bcc29f679e0e	9668	intrahospital transfer events
Lab_dictionary.csv	626f42ef850c43acab8f6ce5f3a51aa9	456	Dictionary for laboratory events
Lab.csv	19846b422711130c43a0cbc6110d9b3f	11082482	Laboratory findings
Medication_Dictionary.csv	f7e5e02e3c8b02a3368c932d29052575	2257	Dictionary for medication events
Medication.csv	0579193671f4a1be2d1c376ac915fd53	1668758	Medication events
MedOrder.csv	282c8e4660ac9e409ef4ed0a2877e52b	1741314	Medical order
MicrobiologyCulture.csv	fea8d113681acf94c375fb5584d29981	242995	Microbiology cuture
NursingChart_IO.csv	ccdd3432431a8c0e29e84beb88f098bf	643641	Fluid Input and output
NursingChart_VitalSign.csv	c211e754c92a78e2a95ffc6229678479	19147518	Vital Sign from Nursing chart
ProgressNote.csv	ac89136f8787b1eb38fcf459a25e5bd9	316299	Progress note during hospitalization
PtAdmiTable.csv	79f2b05a8925a3b8e25aec94c05ee124	8180	Patient admission table
SurgeryTab.csv	189a5854bbeca548167137403b8bfd22	7706	Surgery event
VitalSign.csv	71786caa7c5251fceaddad85e707f419	5565775	Vital signs

### Patient admission record table

The patient admission record table describes the baseline patient demographics, past history, chief complain, and length of stay in the hospital. The *patient_SN* is a unique ID for individual patient and *Hospital_ID* is unique ID for hospital admission. If a patient discharged/died within 24 hours, the data were recorded in a separate table, so there are separate columns describing the chief complain and admission status for those short hospital stays. We provide both English and Chinese descriptions for chief complain. The present history recorded in the *Med_history* column contains more words, and the original Chinese descriptions are kept so that some natural language processing algorithms can be applied. The StatusOnDischarge variable includes several categories such as Cured, Not cured, Unknown and Dead. These categories are recorded as that in the original electronic system. The “Not cured” status refers to the situation when a patient was discharged against medical order and might be transferred to the primary care service center or go home for palliative care. The “Unknown” label is also entered by the clinicians and should be considered as a separate type of status (Table 3).

**Table 3:** variables in the patient admission record table.

Variables	Explanation
patient_SN	Patient series number: unique to each individual subject
Hospital_ID	unique to each hospital admission
Sex	Sex coded as Male and Female
Age_cut	Age categorized into bins for confidentiality
PastHistory	Past history/comorbidities in English. Diseases are separated by semi-coma
ChiefComplain_24hr	Chief Complain about patients who discharged within 24 hours after hospital admission
AdmissionStatus_24hr	Admission Status for patients who discharged within 24 hours after hospital admission
ChiefComplain_24hr_dead	Chief Complain about patients who died within 24 hours after hospital admission
AdmissionStatus_24hr_dead	Admission Status for patients who died within 24 hours after hospital admission
ChiefComplain	Chief Complain in Chinese
Med_history	Medical history in text
StatusOnDischarge	Status On Discharge
DiagnosisOnDeath	Diagnosis On Death
StatusOnDischarge_Desc	Status On Discharge described in text
Discharge_DateTime	Discharge time relative to hospital admission time as the time zero in days
DaysHospitalStay	Days of Hospital Stay
ChiefComplain_Eng	Chief Complain in English

### Electronic medical record (First note table)

The FirstNote.csv table contains data related to the progress note recorded on the admission day (Table 4), which includes free text data such as the reasons for diagnosis, differential diagnosis and care plan. The diagnosis in this table is the initial diagnosis made on the day of admission and is subject modifications.

**Table 4:** variables in the FirstNote table.

Variables	Explanation
patient_SN	Patient series number: unique to each individual subject
Hospital_ID	unique to each hospital admission
FirstNote_DateTime	The date on which the first note is recorded (days relative to the admission time)
FirstNote_DESC	The contents of the first note
DiagnosisReason	The reasons for diagnosis
InitialImpression	The initial diagnosis that might be modified during hospitalization
MedHisSummary	Brief summary of the medical history
DiffDiagnosis	Differential diagnosis
CarePlan	Care plan during the hospitalization

### Progress note table

The progress note table (ProgressNote.csv) contains information on a variety of daily progress notes such as Daily course record, Blood transfusion record, and record for bedside procedures (Table 5).

### Diagnosis table

The diagnosis table contains information related to diagnosis for a hospital stay (Table 6). The *Diagnosis_Desc* column provides free text description for the diagnosis. ICD10_code is the code number for the standard ICD code. The information can be well processed with the *icd* package in R (https://github.com/cran/icd). The functionality of the package includes but not limited to finding comorbidities of patients based on ICD-10 codes, Charlson and Van Walraven score calculations, and comprehensive test suite to increase confidence in accurate processing of ICD codes.

### Hospital transfer table

The *HospitalTransfer* table contains information related to intrahospital transfer events (Table 7). The time and department of each transfer event are given in respective columns. In the table, one row represents one transfer event, including the department a patient leaves (*TransferFrom_Dept_Eng*) and another department a patient transfer into (*TransferTo_Dept_Eng*). One episode of hospitalization may contain multiple transfer events. To protect patients’ privacy, all date and time information is recorded as days relative to hospital admission. Since the EICU is in the emergency department, the department names denoted by “Emergency medical department” or “Emergency Department” refer to the EICU.

**Table 5:** Variables in the ProgressNote table.

Variables	Explanation
patient_SN	Patient series number: unique to each individual subject
Hospital_ID	unique to each hospital admission
Note_DateTime	The time for the note record, in reference to the admission
Note_DESC	Free text descriptions for the note
NoteType	The type of note

**Table 6:** variables in the Diagnosis table.

Variables	Explanation
patient_SN	Patient series number: unique to each individual subject
Hospital_ID	unique to each hospital admission
Diagnosis_Desc	Description of diagnosis in free text
ICD10_code	ICD-10 code
ICD10_name	ICD-10 name for the diagnosis in English
Diagnosis_DateTime	Time for making the diagnosis relative to hospital admission time as the time zero in days

### Surgery information table

The surgical operation information is recorded in a separate table (SurgeryTab.csv). The table records the scheduled time for operation and descriptions for the operation. The name of the operation can be extracted from the text descriptions (Oper_Scheduled). The medical order for a planned operation is usually prescribed 1 day prior to the operation. If the planned date takes a minus value, it can be regarded that the operation is performed on the day of hospital admission (Table 8).

### The Lab table

The lab table contains data related to the laboratory findings (Table 9). There are 11,082,482 records of laboratory items in the dataset involving 214 types of laboratory items. there are 17 types of samples being tested for laboratory findings, including whole blood, plasma, urine, serum, arterial blood, stool, venous blood, catheter orifice, ascites, bile, dialysate, CK blood sample (kaolin-activated TEG channel), cerebrospinal fluid, bone marrow, deep venous catheter, sputum, gastric juice. The sample collection time is also recorded in days in reference to the hospital admission time. The *Lab_category* column may contain missing values for the following reasons: (1) the laboratory category is missing for some laboratory items that are derived from other values, such as INR, Urea: creatinine, and Arterial alveolar oxygen partial pressure ratio; (2) Some laboratory items are exported from the bedside point-of-care machines, such as troponin and blood gas items in an acute care setting; their laboratory category is not integrated into the laboratory system; and (3) some values not directly assayed by the machine such as inspired oxygen saturation (FiO2), and prothrombin time control. Since the missing information in the laboratory category will not influence the research outcome; we did not populate these missing cells.

### The Lab dictionary

To facilitate the use of the Lab table, we generated a lab dictionary table (Table 10) which included the unique names of lab items and the lab category.

### Microbiology culture table

The *MicrobiologyCulture* table contains information related to microbiology culture results (Table 11). Conventional information regarding sample, culture finding, culture time and description of microbiology culture are provided in the table.

**Table 7:** Explanation for variables in the *HospitalTransfer* table.

Variables	Explanation
patient_SN	Patient series number: unique to each individual subject
Hospital_ID	unique to each hospital admission
TransferIn_DateTime	The time of transfer into a department, recorded in days relative to hospital admission
TransferOut_DateTime	The time of transfer out of a department, recorded in days relative to hospital admission
TransferTo_Dept_Eng	The department a patient will arrive (transfer into)
TransferFrom_Dept_Eng	The department a patient will leave (transfer out)

**Table 8:** Explanation for variables in the *SurgeryTab* table.

Variables	Explanation
patient_SN	Patient series number: unique to each individual subject
Hospital_ID	unique to each hospital admission
MedOrder_Type	Type of medical order: regular or stat
MedOrder_Start_DateTime	Start time of medication in days relative to hospital admission
MedOrder_Stop_DateTime	Stop time of medication in days relative to hospital admission
PlanedDate	Planned date for the operation in days relative to hospital admission
Oper_Scheduled	Text descriptions for the scheduled operation

**Table 9:** Explanations for variables in the *Lab* table.

Variables	Explanations
patient_SN	Patient series number: unique to each individual subject
Hospital_ID	unique to each hospital admission
Lab_category	Category of lab item
Lab_DateTime	Time of lab in days relative to hospital admission
Lab_results	Results of the lab finding
Unit_measure	Unit of measurement
LabSampleCollect_time	Sample collection time in days relative to hospital admission
Lab_ItemName	Name of lab item
Lab_SampleName	Sample name

**Table 10:** Dictionary for laboratory items.

Variables	Explanation
Lab_category	Category of lab item
Lab_ItemName	Name of lab item
Lab_SampleName	Sample name
Index	An index column containing distinct values for each row

**Table 11:** Explanation for variables in the Microbiology culture table.

Variables	Explanation
patient_SN	Patient series number: unique to each individual subject
Hospital_ID	unique to each hospital admission
MicrobiologyCulture_Finding	Microbiology Culture finding
MicrobiologyCulture_DateTime	Microbiology Culture time in days relative to hospital admission
MicrobiologyCulture_sample_Eng	Microbiology Culture sample
MicrobiologyCulture_Category_Eng	Microbiology Culture Category
MicrobiologyCulture_DESC_Eng	Description of Microbiology Culture

### Drug sensitivity table

The *DrugSens* table contains information related to the drug sensitivity of cultured bacteria (Table 12). Conventional information including sample, microbiology, culture time, and drug name is available in the table. The negative and positive values in the *DrugSens_result* column refer to the results for Ultra broad spectrum β- Lactamase or D-test.

**Table 12:** The explanation for variables in the Drug sensitivity table.

Variables	Explanation
patient_SN	Patient series number: unique to each individual subject
Hospital_ID	unique to each hospital admission
Drug_Code	Code of the drug for sensitivity analysis
DrugSens_result	Results for Drug Sensitivity test
MIC	Minimum inhibitory concentration
DrugSens_DateTime	Time for the results relative to hospital admission time as the time zero in days
Drug_name_Eng	Name of the tested drug
DrugSens_Microbiology_Eng	Microorganism for testing
DrugSens_Category_Eng	Category for the test
DrugSens_sample_Eng	Sample name

### Examination report table

The *ExamReport* table contains information related to a variety of medical examinations, including computed topography (CT), X-ray and ultrasound (Table 13). The images are not available in current dataset, but instead we include the free text descriptions and conclusions for these examinations.

**Table 13:** Explanation for variables in the *ExamReport* table.

Variables	Explanation
patient_SN	Patient series number: unique to each individual subject
Hospital_ID	unique to each hospital admission
ExamReport_Category	Category of examination
ExamReport_DESC	Description of the examination in free form text
ExamReport_Finding	Result finding
ExamReport_DateTime	Time for the examination results relative to hospital admission time as the time zero in days
ExamReport_item_Eng	Name of the Examination

### Medical order table

The *MedOrder* table contains information related to the medical order prescribed by clinicians (Table 14). The table provides both regular and stat medical orders (*MedOrder_Type*). The contents of the medical order can be found in the *MedOrder_DESC* column.

**Table 14:** Explanation for variables in the *MedOrder* table.

Variables	Explanation
patient_SN	Patient series number: unique to each individual subject
Hospital_ID	unique to each hospital admission
MedOrder_Type	Type of medical order: regular or stat
MedOrder_DESC	Description of medical order in free text
MedOrder_Start_DateTime	Start time of medication in days relative to hospital admission
MedOrder_Stop_DateTime	Stop time of medication in days relative to hospital admission

### Medication table

The medication table provides data on the medication orders prescribed by clinicians (Table 15). This table is designed specifically for medication orders, containing columns for drug dose, frequency, unit of drug dose and route of administration.

**Table 15:** The explanation for variables in the Medication table.

Variables	Explanation
patient_SN	Patient series number: unique to each individual subject
Hospital_ID	unique to each hospital admission
Med_category	Category of medication
SingleDose	Single dose
Med_Freq	Frequency of administration
Med_unit	Unit of measurement
Med_startTime	Start time of medication in days relative to hospital admission
Med_stopTime	Stop time of medication in days relative to hospital admission
Med_route_Eng	Route of administration
Med_DESC_Eng	Medication name in text

### Medication dictionary

The Medication_Dictionary table provides information for the unique medication names. Some medications can be easily obtained from the dictionary table. We provided a DrugName column where users can easily look up unified pharmaceutical names irrespective of the specifications, formula, and route of administration. For example, if we want to extract sodium chloride injection, we can look for sodium chloride in the DrugName column. Alternatively, users may search the Med_DESC_Eng column with the key words “Sodium chloride”. This can be easily achieved by the *stringr* pipeline in R (Table 16).

**Table 16:** Medication dictionary table.

Variables	Explanation
Med_category	Category of medication, Western medicine vs. Chinese traditional medicine
Med_DESC_Eng	Medication name in text
DrugName	Unified pharmaceutical names irrespective of the specifications, formula and route of administration

### Vital sign table

The *VitalSign* table provides vital sign data for each hospital admission (Table 17). The *VitalSign_DESC* column provides categories of vital signs including diastolic blood pressure, temperature, heart rate and respiratory rate.

**Table 17:** Explanation for variables in the *VitalSign* table.

Variables	Explanation
patient_SN	Patient series number: unique to each individual subject
Hospital_ID	unique to each hospital admission
VitalSign_DESC	Vital Sign Description
VitalSign_value	Vital Sign value
VitalSign_unit	Vital Sign unit of measurement
VitalSign_DateTime	Vital Sign measurement time in days relative to hospital admission

## Technical Validation

Data were verified for integrity during the data transfer process from the hospital information system to the database platform using MD5 checksums (Table 2). The MD5_hashes presented in Table 2 can also be used by users to ensure the integrity of the downloaded datasets. All text information extracted from our medical information system are in Chinese. In establishing our data warehouse, we translated some meta-data and short text to facilitate the use of data by researchers outside China. The translation was first performed by using the paid BaiDu academic translation service (service number: MPE2022102608424528825) and then checked by two authors (Senjun Jin and Zhongheng Zhang) of the project. However, in order to maintain data fidelity, very little post-processing has been performed for other long text fields such as present history, progress notes, and text reports of image studies. Some natural language contents were not translated into English because any translations may change the results of natural language processing or text mining^20,21^. Users can employ some academic language translation services (including API) for a large volume of language translation if needed.

The medical data archived within the database were originally not intended for secondary analysis. Thus, some missing values and inconsistencies may occur due to technical errors, system integration, and data preprocessing. In particular, the electronic critical care nursing chart system was launched in the year 2018, and thus the current database contained no information before that time. These older nursing chart data before 2018 are recorded manually and archived in paper documents. We are planning to convert these data into electronic information in a future project.

## Usage Notes

### Data access

Data are deposited in the PhysioNet repository (https://physionet.org/) and can be accessed after completion of an online course (e.g. from the Collaborative Institutional Training Initiative)^22^. Data access also requires a data use agreement to be signed, which stipulates that the user will not try to re-identify any subjects, will not share the data, and will release code associated with any publication using the data. Once approved, the plain CSV files can be directly downloaded from the project on PhysioNet^22^.

## Data Availability

The code for establishing the database was available on GitHub: https://github.com/zh-zhang1984/ZhejiangProvinceICU/blob/main/ZhejiangProvinceICU.md
